# Media Ion Composition Controls Regulatory and Virulence Response of *Salmonella* in Spaceflight

**DOI:** 10.1371/journal.pone.0003923

**Published:** 2008-12-12

**Authors:** James W. Wilson, C. Mark Ott, Laura Quick, Richard Davis, Kerstin Höner zu Bentrup, Aurélie Crabbé, Emily Richter, Shameema Sarker, Jennifer Barrila, Steffen Porwollik, Pui Cheng, Michael McClelland, George Tsaprailis, Timothy Radabaugh, Andrea Hunt, Miti Shah, Mayra Nelman-Gonzalez, Steve Hing, Macarena Parra, Paula Dumars, Kelly Norwood, Ramona Bober, Jennifer Devich, Ashleigh Ruggles, Autumn CdeBaca, Satro Narayan, Joseph Benjamin, Carla Goulart, Mark Rupert, Luke Catella, Michael J. Schurr, Kent Buchanan, Lisa Morici, James McCracken, Marc D. Porter, Duane L. Pierson, Scott M. Smith, Max Mergeay, Natalie Leys, Heidemarie M. Stefanyshyn-Piper, Dominic Gorie, Cheryl A. Nickerson

**Affiliations:** 1 The Biodesign Institute, Center for Infectious Diseases and Vaccinology, Arizona State University, Tempe, Arizona, United States of America; 2 Department of Biology, Villanova University, Villanova, Pennsylvania, United States of America; 3 Habitability and Environmental Factors Division, NASA-Johnson Space Center, Houston, Texas, United States of America; 4 Tulane University Health Sciences Center, New Orleans, Louisiana, United States of America; 5 Flanders Institute of Biotechnology, Free University of Brussels, Brussels, Belgium; 6 Belgian Nuclear Research Center, Mol, Belgium; 7 Sidney Kimmel Cancer Center, San Diego, California, United States of America; 8 Center for Toxicology, University of Arizona, Tucson, Arizona, United States of America; 9 The Biodesign Institute, Center for Glycoscience Technology, Arizona State University, Tempe, Arizona, United States of America; 10 Wyle Laboratories, Houston, Texas, United States of America; 11 NASA-Ames Research Center, Moffett Field, California, United States of America; 12 Space Life Sciences Lab, Kennedy Space Center, Cape Canaveral, Florida, United States of America; 13 BioServe, University of Colorado, Boulder, Colorado, United States of America; 14 School of Medicine, University of Colorado Denver, Aurora, Colorado, United States of America; 15 Oklahoma City University, Oklahoma City, Oklahoma, United States of America; 16 Section of General Surgery, University of Chicago, Chicago, Illinois, United States of America; 17 Departments of Chemistry, Chemical Engineering and Bioengineering, University of Utah, Salt Lake City, Utah, United States of America; 18 Human Adaptation and Countermeasures Division, Johnson Space Center, National Aeronautics and Space Administration, Houston, Texas, United States of America; 19 Astronaut Office, NASA-Johnson Space Center, Houston, Texas, United States of America; Massachusetts General Hospital, United States of America

## Abstract

The spaceflight environment is relevant to conditions encountered by pathogens during the course of infection and induces novel changes in microbial pathogenesis not observed using conventional methods. It is unclear how microbial cells sense spaceflight-associated changes to their growth environment and orchestrate corresponding changes in molecular and physiological phenotypes relevant to the infection process. Here we report that spaceflight-induced increases in *Salmonella* virulence are regulated by media ion composition, and that phosphate ion is sufficient to alter related pathogenesis responses in a spaceflight analogue model. Using whole genome microarray and proteomic analyses from two independent Space Shuttle missions, we identified evolutionarily conserved molecular pathways in *Salmonella* that respond to spaceflight under all media compositions tested. Identification of conserved regulatory paradigms opens new avenues to control microbial responses during the infection process and holds promise to provide an improved understanding of human health and disease on Earth.

## Introduction

The environment of spaceflight offers insight into fundamental cellular and molecular mechanisms directly relevant to human health and infectious disease that cannot be observed using traditional experimental approaches [1], [2]. The recent discovery that microbial pathogens cultured during spaceflight alter their virulence profiles reveals the potential for identification of novel classes of genes and proteins that are critical for the infection process [2]. Although distinct bacterial responses have been repeatedly observed during culture in spaceflight [1], [2] the mechanism(s) that initiates these responses is unclear. Historically, studies of microbial responses in extreme environments such as severe shifts in pH, osmotic concentration, or temperature, have provided tremendous insight into our understanding of how pathogens adapt and respond during the infection process *in vivo* [3], [4]. Many of these conditions reflect the environment that pathogens encounter during the normal course of infection [4], [5]. In this regard, the conditions that microorganisms experience in the microgravity environment of spaceflight are relevant to low fluid shear areas encountered by pathogens during infection of a human host, such as between brush border microvilli in the intestinal tract [6], [7], [8]. This latter environment holds particular importance for enteric pathogens such as *Salmonella*.

In our early studies, *Salmonella enterica* serovar Typhimurium (hereafter referred to as *S. typhimurium* or *Salmonella*) was cultured in a ground-based bioreactor that was used to model the low fluid shear culture conditions that occur in microgravity as well as in certain *in vivo* environments. This device, known as a rotating wall vessel (RWV) bioreactor, produces an environment in which the bacteria are exposed to low fluid shear during culture (Figure S1) [1], [9]. When *S. typhimurium* was cultured in this manner using Lennox Broth (LB) media, the organism exhibited increased virulence in a murine model of infection [9], [10], increased resistance to environmental stresses [9], [10], and differentially expressed 163 genes [11]. In particular, the Ferric Uptake Regulator (Fur) protein was shown to play a role in low fluid shear induced acid stress resistance [11]. Evaluation of microarray data from *Salmonella* cultures grown in the RWV indicated that a large number of genes encoding ion response pathways were differentially regulated in response to low fluid shear growth conditions [11]. In addition, the potential role for ion-mediated regulation of cellular responses to this environment was also observed in additional RWV bioreactor experiments, showing that in M9 minimal media, *Salmonella* lag phase and generation time were shortened compared to controls; an effect not observed in LB media under identical growth conditions [10]. The influence of media composition observed with bacteria grown in the RWV bioreactor is not solely restricted to *S. typhimurium*. A comparison of the gene expression profiles from *Escherichia coli* cultures grown in the RWV revealed distinct differences in the genes that were differentially expressed when cultured in either MOPS minimal media or LB media [12].

These observations led to flight experiments with *S. typhimurium* aboard Space Shuttle mission STS-115 (September 2006), which demonstrated that cultures grown in rich LB media in spaceflight displayed significant alterations in global gene expression, increased virulence, and biofilm-like formation as compared to ground controls [2]. A central role was identified for the conserved, small regulatory RNA-binding protein Hfq in regulating key aspects of the spaceflight microgravity response [2]. In addition, many genes encoding ion response pathways showed altered expression during spaceflight, reinforcing our earlier results observed in the spaceflight-analogue RWV bioreactor culture of *S. typhimurium*. These results led us to hypothesize that media ion concentrations could be manipulated to prevent the enhanced *Salmonella* virulence imparted during spaceflight.

To test this hypothesis and further understand the mechanistic effect of spaceflight on bacterial virulence, we performed analyses on *Salmonella* cultured in varying media conditions aboard Space Shuttle missions STS-115 and STS-123. In each case, the flight culture samples were compared to culture samples grown under identical conditions on the ground at Kennedy Space Center using coordinated activation and termination times (via real time communications with the Shuttle crew) in an insulated room that maintained the identical temperature and humidity as that on the Shuttle (Orbital Environment Simulator or OES). The culture experiments were loaded into specially-designed hardware (termed fluid processing apparatus or FPA) to facilitate controlled activation and fixation of the cultures while maintaining suitable culture containment requirements (Figure S3). These experiments allowed the identification of a) media ion composition that prevents spaceflight-induced increases in *Salmonella* virulence, and b) commonalities and differences in *Salmonella* gene expression between cultures of the same pathogen in different media during spaceflight. As with spaceflight growth in LB, we show here that *Salmonella* grown in M9 media during flight displayed differential expression of many genes, including those associated with either the regulation of, or regulation by, the Hfq protein and small regulatory RNAs. Moreover, *Salmonella* grown in various media demonstrated that ion concentrations had a direct effect on the virulence of the spaceflight cultures. Specifically, we show that media ion composition can be used to prevent the enhanced *Salmonella* virulence imparted during spaceflight. Furthermore, we show that higher concentrations of phosphate ions present in M9 medium during spaceflight analogue culture in the RWV altered its pathogenic-related effects, thus providing the first evidence of a mechanism behind this response. These findings indicate that virulence in *Salmonella* can be influenced by the environmental stimulus of spaceflight, and that the response to this stimulus can be manipulated to improve astronaut health measures and exploited to better understand microbial pathogenesis and develop innovative therapeutics.

## Results

### Media and virulence in spaceflight–LB

**Figure 1:**
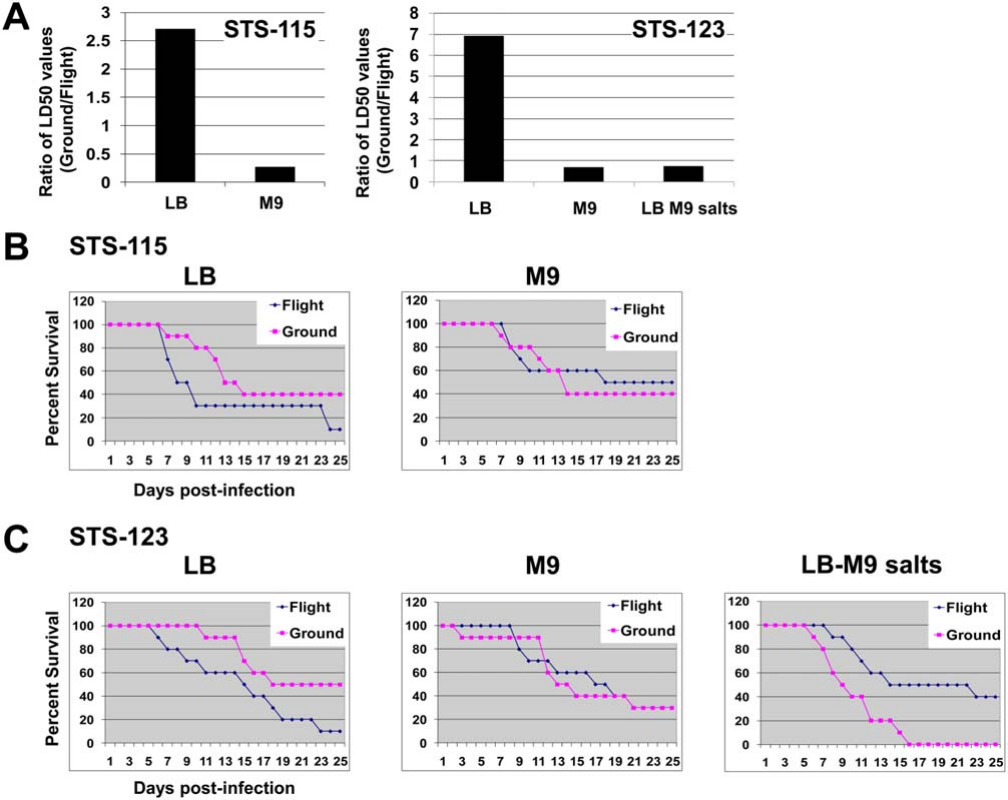
*S. typhimurium* virulence in LB, M9 and LB-M9 spaceflight cultures. A) Ratio of LD_50_ values of *S. typhimurium* spaceflight and ground cultures grown in LB (Lennox Broth), M9, or LB-M9 salts media. Female Balb/c mice were perorally infected with a range of bacterial doses from either spaceflight or ground cultures and monitored over a 30-day period for survival. B) Time-to-death curves of mice infected with spaceflight and ground cultures from STS-115 (infectious dosage: 10^7^ bacteria for both media). C) Time-to-death curves of mice infected with spaceflight and ground cultures from STS-123 (infectious dosage: 10^6^ bacteria for LB and 10^7^ bacteria for M9 and LB-M9 salts). Infectious dosages were selected such that the rates in time-to-death facilitated normalized comparisons across the different media.

Our previous flight experiment aboard STS-115 indicated that *S. typhimurium* cultured during spaceflight exhibited increased virulence in a murine model of infection [2]. Briefly, bacteria cultured in LB during spaceflight and identical ground control cultures were harvested and immediately used to inoculate female Balb/c mice via a per-oral route of infection on the same day as Shuttle landing. Mice were infected at increasing dosages of either flight or ground cultures (10 mice per dose), and the health of the mice was monitored every 6–12 hours for 30 days. Our previous results showed that mice infected with *S. typhimurium* grown in LB media in spaceflight aboard STS-115 displayed a decreased time-to-death and a 2.7 fold decrease in LD_50_ value compared with those infected with ground control cultures [2]. To confirm these findings, the identical flight experiment was performed again aboard STS-123 (Figures S2 and S3). In agreement with the previous experiment, mice infected with *S. typhimurium* grown in spaceflight aboard STS-123 displayed a decreased time-to-death and a 6.9 fold decrease in LD_50_ value compared with those infected with ground control cultures (Figure 1, panels A, B, and C, LB medium).

### Media and virulence in spaceflight–M9

Because of the strong association between nutrient composition of the growth media and the extent of changes observed in *S. typhimurium* responses in ground-based studies in the RWV, we evaluated *S. typhimurium* virulence using cultures grown in M9 minimal media in separate experiments aboard Space Shuttle missions STS-115 and STS-123. The procedures were otherwise identical to those described for LB media growth [2]. Interestingly, M9 cultures from both missions displayed dramatically different virulence characteristics from those observed for *Salmonella* cultured in LB (Figures 1A, 1B and 1C). Specifically, for infection of mice with spaceflight and ground control *Salmonella* cultures grown in M9 media, the time-to-death curves overlapped and did not display the decrease in time-to-death as seen in the LB spaceflight infections in both STS-115 and STS-123. Likewise, in contrast with the decrease in LD_50_ values observed for the LB flight cultures as compared to ground controls, M9 cultures of *S. typhimurium* grown in spaceflight displayed no consistent difference in LD_50_ from ground controls.

To further elucidate the effect of media composition on the virulence characteristics of *S. typhimurium* grown during spaceflight, an additional growth medium was used that consisted of LB media supplemented with specific salts used in the preparation of M9 media. These specific salts were chosen because our quantitative trace elemental analysis showed them to be at significantly different levels in the two media. Specifically, the elemental analysis indicated that the M9 medium had dramatically higher concentrations of phosphate (61-fold higher than the LB media) and magnesium (18-fold higher than the LB media). Other significant differences in the M9 medium included higher levels of sulfate (3.6-fold higher than the LB media), chloride (3-fold higher than the LB media), and potassium (2.4-fold higher than the LB media). Thus, for our follow-up flight experiment aboard STS-123, we evaluated *S. typhimurium* virulence using cultures grown in LB media supplemented with 25.18 mM NaH_2_PO_4_, 22 mM KH_2_PO_4_, 18.68 mM NH_4_Cl, 8.54 mM NaCl, and 2 mM MgSO_4_ (designated as LB-M9 salts media), thereby bringing the levels of these salts in LB media to the same as those in M9 media. Interestingly, *Salmonella* cultured in LB-M9 salts media displayed virulence characteristics similar to those observed when only the M9 media was used (Figures 1A and 1C). Specifically, as seen with cultures grown in only M9 media, mice infected with spaceflight and ground cultures grown in LB-M9 salts media did not display the decrease in time-to-death with spaceflight-grown cultures as observed in the LB infections. Also in contrast to the LB media cultures, cultures of *S. typhimurium* grown in LB-M9 salts media during spaceflight did not display a decreased LD_50_ value compared to ground controls using the same media (similar to the results with M9 media).

Since nutrient composition could influence the virulence of *S. typhimurium* [13], we compared the LD_50_ values for all media from flight and all media from ground controls from the STS-123 mission to highlight the effect of spaceflight on virulence (Table 1). A comparison of LD_50_ values from ground controls suggests that indeed media plays a role in LD_50_ levels, with a 5.7 fold difference between LB media and M9 media (with LB showing lower LD_50_ values). However, a comparison of LD_50_ values of cultures grown during spaceflight shows a dramatic difference approximately 10 times greater than those observed in ground cultures, as shown with a 56.8 fold difference between LB media and M9 media. This difference suggests that while media composition does affect LD_50_ values, the difference is exacerbated by the spaceflight environment.

**Table 1:** LD_50_ comparison of *S. typhimurium* cultured in M9 media or LB-M9 salts media relative to cultures grown only in LB media.

Media	Growth Location	LD_50_ (CFU)	Fold Increase Relative to LB Media - Flight
LB media	Flight	5.81×10^4^	1.0
LB-M9 salts media	Flight	7.45×10^5^	12.8
M9 media	Flight	3.30×10^6^	56.8

Media	Growth Location	LD_50_ (CFU)	Fold Increase Relative to LB Media - Ground
LB Media	Ground	4.02×10^5^	1.0
LB-M9 salts media	Ground	5.73×10^5^	1.4
M9 media	Ground	2.30×10^6^	5.7

### Transcriptional and proteomic analysis

To determine which *Salmonella* genes changed expression in response to spaceflight culture in M9 minimal media, total bacterial RNA was isolated from fixed flight and ground samples, qualitatively analyzed to ensure lack of degradation, quantified, and then reversed transcribed into labeled, single-stranded cDNA. The labeled cDNA was co-hybridized with differentially-labeled *S. typhimurium* genomic DNA to whole genome *S. typhimurium* microarray slides. Statistically-significant differences in gene expression between the flight and ground M9 samples (above 1.8-fold increase and below 0.6-fold decrease in expression) were obtained (see Materials and Methods for details). We found 38 genes differentially-expressed in flight M9 cultures as compared to identical ground controls under these conditions (Table 2). Most notably, several genes involved in motility (9 genes: *flgA*, *flgC*, *flgF*, *flgG*, *cheY*, *fliC*, *fliT*, *fliM*, *fljB*), the formation of the Hyc hydrogenase (4 genes: *hydN*, *hycF*, *hycD*, *hyB*), and the Suf membrane transporter (3 genes: *sufA*, *sufC*, *yhnA/sufE*) were identified as differentially expressed. In addition, several genes encoding small regulatory RNA molecules (THI, *csrB*, *rnpB*, *tke1*) were also identified.

**Table 2:** *Salmonella typhimurium* genes altered in expression during growth in M9 minimal media in spaceflight.

Up-regulated				
STM gene	Fold change	Identified in LB analysis*	Gene name	Gene function
STM_sRNA_THI	2.69	x	*THI* **	small RNA
STM0007	1.91		*talB*	transaldolase B
STM0389	1.85	x	*yaiA*	putative cytoplasmic protein
STM1161.S	2.64		*yceP*	putative cytoplasmic protein
STM1369	2.81	x	*sufA*	putative HesB-like domain
STM1371	2.65	x	*sufC*	putative ABC superfamily (atp_bind) transport protein
STM1374	1.84	x	*ynhA*	putative SufE protein probably involved in Fe-S center assembly
STM1724	1.96	x	*trpD*	anthranilate synthase, component II, bifunctional
STM2665	2.53	x	*yfiA*	ribosome associated factor, stabilizes ribosomes against dissociation
STM2924	2.55		*rpoS*	sigma S (sigma 38) factor of RNA polymerase
STM3347	1.83	x	*yhcB*	putative periplasmic protein
STM3559	2.05		*yhhV*	putative cytoplasmic protein
STM3809.S	1.83		*ibpA*	small heat shock protein
STM4161	2.00			putative involved in thiamine biosynthesis
**Down-regulated**				
STM_PSLT014	0.52		*orf6*	putative outer membrane protein
STM_sRNA_CsrB	0.51	x	*csrB*	regulatory RNA
STM_sRNA_RNaseP	0.44	x	*rnpB*	regulatory RNA
STM_sRNA_tke1	0.58	x	*tke1*	small RNA
STM1078	0.43			putative cytoplasmic protein
STM1165	0.57	x	*grxB*	glutaredoxin 2
STM1173	0.57	x	*flgA*	flagellar biosynthesis; assembly of basal-body periplasmic P ring
STM1175	0.37	x	*flgC*	flagellar biosynthesis, cell-proximal portion of basal-body rod
STM1178	0.52	x	*flgF*	flagellar biosynthesis, cell-proximal portion of basal-body rod
STM1179	0.47	x	*flgG*	flagellar biosynthesis, cell-distal portion of basal-body rod
STM1196	0.59	x	*acpP*	acyl carrier protein
STM1466	0.59		*ydgA*	putative periplasmic protein
STM1916	0.55	x	*cheY*	chemotaxis regulator, transmits chemoreceptor signals to flagellar motor
STM1959	0.44	x	*fliC*	flagellar biosynthesis; flagellin, filament structural protein
STM1962	0.54	x	*fliT*	flagellar biosynthesis; possible export chaperone for FliD
STM1976	0.59	x	*fliM*	flagellar biosynthesis, component of motor switch and energizing
STM2646	0.44	x	*yfiD*	putative formate acetyltransferase
STM2771	0.31	x	*fljB*	Flagellar synthesis: phase 2 flagellin (filament structural protein)
STM2843	0.49	x	*hydN*	electron transport protein (FeS senter) from formate to hydrogen
STM2848	0.59	x	*hycF*	hydrogenase 3, putative quinone oxidoreductase
STM2850	0.59	x	*hycD*	hydrogenase 3, membrane subunit (part of FHL complex)
STM2852	0.52	x	*hycB*	hydrogenase-3, iron-sulfur subunit (part of FHL complex)
STM4002	0.53	x		putative cytoplasmic protein
STM4063	0.55		*sbp*	ABC superfamily (bind_prot), sulfate transport protein

* Genes, operons, or directly-related functional groups identified as also being differentially-regulated during growth in spaceflight or ground-based modeled microgravity in LB medium.

** STM genome coordinates: 4382782 - 4382542.

The proteomes of fixed cultures from M9 flight and ground samples were also obtained via multi-dimensional protein identification (MudPIT) analysis. We identified 173 proteins expressed in the flight and ground cultures, with 81 being present at statistically different levels in these samples (Table S1) indicating differential expression or stability. Notably, several proteins involved in iron utilization and uptake (Fur, cytoplasmic ferritin, Fe-S cluster formation, bactoferrin, siderophore receptor TonB, iron transport protein, iron-dependent alcohol dehydrogenase, and ferric enterobactin receptor) and ribosome structure (L7, L32, S20, S13, S11 S19, L14, L33, S4, L4) were identified as differentially expressed. Collectively, these transcriptional and proteomic gene expression changes form the first documented bacterial spaceflight stimulon in minimal growth media.

### The LB and M9 spaceflight stimulons

We compared the *S. typhimurium* gene expression data from the analysis above in M9 medium to the results from our previous gene expression analysis in LB medium for spaceflight [2] and RWV cultures [1]. Genes from each data set were cross-compared to each other to identify common genes that were present as differentially-expressed in both media. After this analysis, we found that 15 genes (including adjacent genes) of the 38 identified as transcriptionally altered in response to spaceflight in M9 medium were also identified as differentially expressed in either spaceflight or ground-based microgravity analogue RWV culture in LB medium. This represents 39% (15/38) of the total genes found in the M9 transcriptional analysis.

We subsequently extended this analysis to include genes that also belong to the same directly-related functional or regulatory gene group (i.e. not necessarily the same gene or operon, but genes that function or are regulated as part of the same mechanism such as motility), and discovered that the percentage of common genes between analysis in M9 and LB media was 73% (28/38) (Table 2). The functional groups of genes that we identified as regulated by spaceflight or ground-based spaceflight analogue culture in both M9 and LB media included those involved in flagellar-based motility, Hyc hydrogenase formation, Suf transporter formation and other ABC transporters, and small regulatory RNA molecules (genes indicated in the section above). Additionally, there are also eight “stand alone” genes that, to our knowledge, are not co-regulated with these gene groups and include four genes encoding putative, uncharacterized proteins (*yaiA*, *trpD*, *yfiA*, *yhcB*, *grxB*, *acpP*, *yfiD*, STM4002). Several genes encoding proteins identified in the spaceflight and ground proteomic analysis of M9 cultures were also identified in the gene expression analysis of M9 and LB cultures as well (Table S1).

Results from our previous studies indicated that 32% of the *S. typhimurium* genes identified as differentially regulated in spaceflight in LB medium belonged to a regulon of genes controlled by the conserved RNA-binding protein Hfq [2]. We also demonstrated that *hfq* was required for alterations in *Salmonella* acid resistance and macrophage survival in response to the RWV ground-based microgravity analogue model [2]. Therefore, we scanned the results of our spaceflight M9 microarray and proteomic analysis for members of a regulon of genes whose expression and activity is regulated by or regulates Hfq, or whose protein products form a functional regulatory complex with Hfq [14], [15], [16], [17]. Consistent with our previous observations in LB, we found four small non-coding regulatory RNA genes (THI, *csrB*, *rnpB*, *tke1*) and three mRNA transcripts (*rpoS*, *sufE*, *fliC*) regulated by Hfq in our microarray analysis in M9 media (7 of 38 or 18%).

When we scanned the hits from our proteomic analysis for relationships to the Hfq regulon, we found 28 of the 81 proteins (34%) that were differentially expressed in response to spaceflight in M9 media belonged to the Hfq regulon, or are part of a directly related functional group of proteins that are regulated by Hfq. Several observations led to the Hfq regulon members being highlighted in our M9 proteomic analysis: 1) Hfq promotes the expression of a large class of ribosomal structural proteins, and we found differential expression of several of these genes in spaceflight (L7/L12, L32, S20, S13, S11, S19, SA, L14, L33, S4, L4); 2) Hfq regulates the expression of the Fur protein and other genes involved in iron metabolism, and we found that Fur and other iron-related genes are differentially regulated by spaceflight in M9 medium (Fur, Dps, NifU, FepA); 3) Several other proteins encoded by genes belonging to the Hfq regulon were also found in this analysis: NmpC, Tpx, PtsI, PtsH, SucC, LeuB, CysP, DppA, OppA, RpoZ, CsrA, RpoB, NlpB. This data, taken together with the microarray data, indicates the commonalities of the spaceflight response in *Salmonella* in both LB and M9 media, and represents the first common genes that have been identified to be regulated by spaceflight and/or ground based spaceflight analogue culture in both rich and minimal media.

### Real time PCR analysis

**Figure 2:**
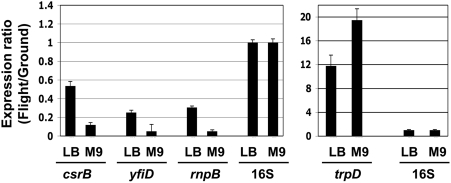
qRT-PCR analysis confirms *S. typhimurium* gene expression altered during spaceflight in LB and M9 media. Total RNA harvested from spaceflight and ground cultures in the indicated media was converted to single-stranded cDNA and used as a template in qRT-PCR analysis with primers hybridizing to the indicated genes. PCR product levels were normalized to the 16S rRNA product and a ratio of each gene level in flight and ground cultures was calculated. All differences in expression between spaceflight and ground cultures were found to be statistically significant using student's t-test (p-value<0.05). The error bars represent the standard deviation for three to nine independent technical replicate experiments.

To further confirm the commonalities we observed in global gene expression analysis in response to spaceflight in both LB and M9 media, we performed targeted quantitative real time PCR assays using cDNA synthesized from total RNA harvested from spaceflight and ground cultures in LB and M9 media as templates (Figure 2). The *csrB*, *yfiD*, *rnpB* genes (down-regulated), and the *trpD* gene (up-regulated) were found to be differentially-regulated in response to spaceflight as compared to ground cultures in both LB and M9 media using global transcriptional analysis. These results were also found using real time PCR (Figure 2) and confirm data from our global analysis.

### Role of phosphate ion

**Figure 3:**
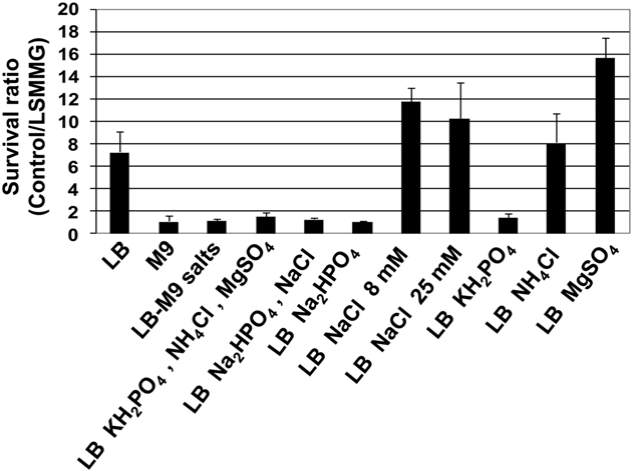
Increased phosphate ion concentration prevents altered *S. typhimurium* acid tolerance in ground-based spaceflight analog culture. Cultures of *S. typhimurium* grown in the indicated medium in the rotating wall vessel in the low-shear modeled microgravity (LSMMG) or control orientation were subjected to acid stress (pH 3.5) immediately upon removal from the apparatus. A ratio of percent survival of the bacteria cultured at each orientation in each media is presented. The error bars represent the standard deviation for two to five independent experimental trials each plated in triplicate. All differences in survival ratios were found to be statistically significant at p-value<0.05.

We previously demonstrated that *Salmonella* consistently and reproducibly alters its acid tolerance response when grown in the RWV using LB medium [9]. To support findings from spaceflight that the supplementation of LB media with selected M9 salts disrupts *S. typhimurium* responses to this envir onment, cultures containing LB media, M9 media, and LB-M9 salts media were grown in the RWV at low shear modeled microgravity (LSMMG/spaceflight analogue culture conditions) and control orientations (Figure S1) and evaluated for changes in acid tolerance. As demonstrated previously, cultures of *S. typhimurium* grown in LB media in the RWV (LSMMG) displayed altered acid resistance as compared to control cultures. However, no difference in acid tolerance was observed with cultures grown in M9 media or in LB-M9 salts media (Figure 3). We then used LB media supplemented with different combinations of M9 salts to determine which of these ions was responsible for disruption of the acid tolerance response observed in LB medium (Figure 3). Our results indicate that the presence of phosphate from two different sources (NaH_2_PO_4_ and KH_2_PO_4_) is sufficient to disrupt the altered acid tolerance in response to LSMMG. Although hydrogen ions are present in each of these compounds, we found no correlation between the pH of the different media before or after culture and the observed phenotypes. Likewise, this finding indicates that the buffering capacity of phosphate is not responsible for this phenotype and that the presence of the phosphate ion itself is responsible for the acid tolerance alteration. In addition, increased osmolarity of the media is not the cause of this phenotype, since raising the level of NaCl to 25 mM (the same level as Na_2_HPO_4_ and KH_2_PO_4_) did not show the same phenotype as the presence of the phosphate-containing compounds (Figure 3).

## Discussion

Results from our initial spaceflight study raised compelling questions regarding the effect of spaceflight on microbial virulence and gene expression [2]. However, given the significant technological and logistical constraints associated with spaceflight experiments, acquiring biological replicates and testing new hypotheses is extraordinarily difficult. Independent experiments conducted on two separate Space Shuttle missions allowed us to establish that spaceflight increases *S. typhimurium* virulence in LB medium. This finding indicates that the effects of spaceflight on *S. typhimurium* are not due to DNA mutations caused by ionizing radiation, since random genomic mutations induced by radiation would not likely be reproduced in multiple experiments. These multiple spaceflight opportunities allowed us to test the hypothesis that changes in ion levels in growth media can serve to disrupt the unique environmental signals of spaceflight sensed by *S. typhimurium* cultures, thereby leading to alterations in microbial virulence. Consistent with our hypothesis, we found that the increased *S. typhimurium* virulence observed with cultures grown in spaceflight in LB medium, as compared to identical ground controls, is not exhibited with cultures grown in M9 medium. Based upon our quantified differences in ion concentrations between LB and M9 media, we supplemented LB medium with inorganic ions to the same levels as those found in M9 medium. We found that this ion supplementation was sufficient to prevent the enhanced *Salmonella* virulence imparted during flight. Subsequent testing in ground-based spaceflight analogue culture conditions indicted that the altered acid tolerance exhibited by *Salmonella* during culture in LB alone was prevented with the addition of inorganic phosphate. These results demonstrate a direct correlation between phosphate ion concentration and the phenotypic response of *Salmonella* to the environment of spaceflight analogue culture.

The correlation of this finding to the actual *in vivo* infection process on Earth is unclear, but there are reports in the published literature that suggest possible clues. Previous studies indicate a relationship between the regulation of phosphate levels *in vivo* and intestinal function, including in response to infection [18], [19], [20], [21], [22], [23]. This relationship could be associated, in part, with the enzyme alkaline phosphatase, which is prevalent in the small intestine and is found on the apical brush border surface. It is known that infection by intestinal pathogens (including *Salmonella*) or their components can significantly alter levels of alkaline phosphatase in the intestine [20], [21], [22], [23]. Moreover, the low fluid shear conditions found near the brush border of the intestine may be similar to the low fluid shear environment of microgravity during spaceflight and could potentially initiate similar *Salmonella* response profiles to those observed in our study [8]. It is also interesting to note that *Salmonella* uses the phosphatase activity of the bacterial enzyme SopB to promote its growth and survival inside intestinal epithelial cells [19]. Taken together, this information suggests a potential relationship between phosphate levels, a low fluid shear environment, and the *Salmonella* enteric infection process *in vivo*, although the specific mechanism(s) is not known.

We also compared the spaceflight-induced molecular genetic responses of *S. typhimurium* cultured in different growth media (LB [2] versus M9 [this work]) using whole genome transcriptional and proteomic analyses. Despite the multiple phenotypic differences in response to spaceflight between the two media, we found that several common genes and gene families were altered in expression in both media during spaceflight culture. Identification of these genes, whose expression is commonly regulated by the low fluid shear environment of spaceflight, provides key targets whose expression can be manipulated to control microbial responses, including potential use for development of vaccines and therapeutics. As identified in this study, these targets include gene systems involved in flagellar-based motility, Hyc hydrogenase formation, Suf transporter formation and other ABC transporters, ribosomal structure, iron utilization, and small regulatory RNA molecule expression and function. Many of the genes that were found differentially expressed during spaceflight culture of *S. typhimurium* in M9 media in this work were also consistent with those reported in LB culture for this same organism under identical conditions [2]. In both cases, many of these genes are found in regulons that are controlled by or regulate the activity of the Hfq protein. Interestingly, Hfq activity has previously been associated with phosphate regulation [24], [25]. Our findings further highlight Hfq as a global regulator to target for further study to understand the mechanism used by *Salmonella* to respond to spaceflight, spaceflight analogue systems, and other physiological low fluid shear environments.

In conclusion, results from these studies support a model in which modulation of different ion concentrations controls the spaceflight-associated virulence response of microorganisms. Moreover, since the low fluid shear environment of spaceflight and spaceflight analogue culture systems is relevant to that encountered by pathogens during the infection process *in vivo*, the identity of commonly conserved molecular targets offers insight for new treatment and prevention strategies. Potential applications of this finding hold exciting promise for mitigating risk to crew health during spaceflight and can be exploited to develop new strategies to combat infectious disease on Earth.

## Materials and Methods

### Strains, media, and chemical reagents

The virulent, mouse-passaged *Salmonella typhimurium* derivative of SL1344, termed χ3339, was used in all flight and ground-based experiments [26]. Lennox broth (LB) (10 g tryptone, 5 g yeast extract, 5 g NaCl) [27], M9 medium (0.4% glucose) [10], or LB - M9 salts medium were used as the growth media in all experiments and phosphate buffered saline (PBS) (Invitrogen, Carlsbad, CA) was used to resuspend bacteria for use as inoculum in the flight hardware. The LB-M9 salts medium consisted of LB medium supplemented with the following amounts of ions: 8.54 mM NaCl, 25.18 mM NaH_2_PO_4_, 18.68 mM NH_4_Cl, 22 mM KH_2_PO_4_, and 2 mM MgSO_4_. The RNA fixative RNA Later II (Ambion, Austin, TX), was used to preserve nucleic acid and protein in flight experiments.

### Spaceflight culture

Spaceflight and ground control cultures were grown in specialized hardware termed fluid processing apparatus (FPA) as described previously [2] (Figures S2 and S3). Briefly, an FPA consists of a glass barrel that can be divided into compartments via the insertion of rubber stoppers and a lexan sheath into which the glass barrel is inserted. Each compartment in the glass barrel was filled with a solution in an order such that the solutions would be mixed at specific time points in flight via two actions: (1) downward plunging action on the rubber stoppers and (2) passage of the fluid in a given compartment through a bevel on the side of the glass barrel such that it was released into the compartment below. Glass barrels and rubber stoppers were coated with a silicone lubricant (Sigmacote, Sigma, St. Louis, MO) and autoclaved separately before assembly. A stopper with a gas exchange membrane was inserted just below the bevel in the glass barrel before autoclaving. FPA assembly was performed aseptically in a laminar flow hood in the following order: 2.0 ml media (either LB, M9 or LB-M9) on top of the gas exchange stopper, one rubber stopper, 0.5 ml PBS containing bacterial inoculum (approximately 6.7×10^6^ bacteria), another rubber stopper, 2.5 ml of either RNA fixative (for gene expression analysis) or media (either LB, M9 or LB-M9 for virulence studies), and a final rubber stopper. Syringe needles (gauge 25 5/8) were inserted into rubber stoppers during this process to release air pressure and facilitate assembly. To facilitate group activation of FPAs during flight and to ensure proper containment levels, sets of 8 FPAs were loaded into larger containers termed group activation packs (GAPs). After activation, cultures were grown for 25 hours in either spaceflight or ground until either fixation or media supplementation. Upon landing, cultures were received for processing approximately 2.5 hours after Shuttle touchdown.

### Microarray analysis

Total cellular RNA purification from cultures grown in M9 media, preparation of fluorescently-labeled, single stranded cDNA probes, probe hybridization to whole genome *S. typhimurium* microarrays, and image acquisition was performed as previously described [2], [11] using three biological and three technical replicates for each culture condition. Direct microscopic cell counting and spectrophotometric readings indicated that cell numbers in flight and ground biological replicate cultures differed by less than 2-fold (data not shown). Data analysis was performed using software as described previously [2]. To obtain the genes comprising the spaceflight stimulon in M9 media, the following parameters were used in Webarray software [28]: an expression ratio of flight to ground of 1.8 fold or greater or 0.6 or less; a spot quality (A-value) of greater than 9.5, and p-value of less than 0.05. To identify spaceflight stimulon genes also contained in the Hfq regulon, proteins or genes found to be regulated by Hfq or RNAs found to be bound by Hfq as reported in the indicated references were scanned against the spaceflight microarray data for expression changes within the parameters above [14], [15], [16], [17]. The microarray data reported in this paper have been deposited in the Gene Expression Omnibus (GEO) database, www.ncbi.nlm.nih.gov/geo (accession no. GSE8573).

### Multidimensional protein identification (MudPIT) analysis via tandem mass spectrometry coupled to dual nano-liquid chromatography (LC-LC-MS/MS)

Acetone-protein precipitates from whole cell lysates obtained from flight and ground cultures grown in M9 media (representing three biological replicates) were subjected to MudPIT analysis using the LC-LC-MS/MS technique (three technical replicates) as described previously [2], [29], [30]. Tandem MS spectra of peptides were analyzed with TurboSEQUEST™ v 3.1 and XTandem software, and the data were further analyzed and organized using the Scaffold program [2], [29], [30]. Please refer to Table S1 for the specific parameters used in Scaffold to identify the proteins in this study.

### Quantitative Real time PCR (qRT-PCR)

qRT-PCR analysis was performed with primers hybridizing to the indicated genes as described previously using the 16S rRNA gene to normalize samples [31]. Data from three to nine separate technical replicate reactions was used for each gene in Figure 2, and the differences in expression were found to be statistically significant using student's t-test (p-value<0.05). The sequences of the primers used here are given in Figure S4.

### Murine infection assay

Six to eight week old female Balb/c mice (housed in the Animal Facility at the Space Life Sciences Lab at Kennedy Space Center) were deprived of food and water for approximately 6 hours and then perorally infected with increasing dosages of *S. typhimurium* harvested from either flight or ground FPA cultures and resuspended in buffered saline gelatin [2]. Infectious dosages increasing ten-fold in a range between approximately 1×10^4^ and 1×10^9^ bacteria (thus comprising six infectious dosages per bacterial culture) were used in the infections. Ten mice per infectious dosage were used, 20 µl per dose, and food and water were returned to the animals within 30 minutes post-infection. The infected mice were monitored every 6–12 hours for 30 days. The LD_50_ value was calculated using the formula of Reed and Muench [32].

### Trace element analysis using Inductively Coupled Plasma (ICP) spectrometry and Ion Chromatography (IC)

Determination of inorganic ion levels in LB and M9 media was performed using ICP spectrometry and IC as described previously [33].

### Ground based RWV cultures and acid stress assays

*S. typhimurium* cultures were grown in rotating wall vessels for 24 hours at 37 degrees C in the LSMMG and control orientations in LB, M9 or LB media supplemented with the indicated ions from M9 salts (LB-M9 salts media) and assayed for resistance to pH 3.5 as described previously [2], [9]. The percentage of surviving bacteria present after 45–60 minutes acid stress (compared to the original number of bacteria before addition of the stress) was calculated via serial dilution and CFU plating. A ratio of the percent survival values for the LSMMG and control cultures in all three growth media was obtained (indicating the fold difference in survival between these cultures) and is presented as the acid survival ratio in Figure 3. The mean and standard deviation from between two and five independent experimental trials per culture is presented with observed differences in survival ratios being statistically-significant at p-value<0.05.

## Supporting Information

Figure S1 The rotating wall vessel (RWV) bioreactor and power supply. Panel A: The cylindrical culture vessel is completely filled with culture medium through ports on the face of the vessel and operates by rotating around a central axis. Cultures are aerated through a hydrophilic membrane that covers the back of the cylinder. The power supply is shown below the bioreactor. Panel B: The two operating orientations of the RWV are depicted. In the LSMMG orientation (panel i), the axis of rotation of the RWV is perpendicular to the direction of the gravity force vector. In the control orientation (panel ii), the axis of rotation is parallel with the gravity vector. Panel C: The effect of RWV rotation on particle suspension is depicted. When the RWV is not rotating, or rotating in the control orientation (panel i), the force of gravity will cause particles in apparatus to sediment and eventually settle on the bottom of the RWV. When the RWV is rotating in the LSMMG position (panel ii), particles are continually suspended in the media. The media within the RWV rotates as a single body, and the sedimentation of the particle due to gravity is offset by the upward forces of rotation. The result is low shear aqueous suspension that is strikingly similar to what would occur in true microgravity.
(0.15 MB PDF)

Figure S2 Experimental setup for STS-115 and STS-123 Salmonella typhimurium microarray and virulence experiments. This flowchart displays a timeline of how the STS-115 and STS-123 experiments were designed and organized. Fluid processing apparatuses (FPAs) were loaded as in Supplemental Figure 3 and delivered to Shuttle, activated during spaceflight, and recovered upon landing as outlined in the flowchart. For a more detailed description of the FPA activation and fixation/supplementation steps, please refer to Supplemental Figure 3. OES: Orbital Environmental Simulator (this is a climate-controlled room at Kennedy Space Center that houses ground controls and is maintained at the same temperature and humidity as the Space Shuttle via real-time communications). SLSL: Space Life Sciences Lab.
(0.01 MB PDF)

Figure S3 Diagram and photographs of fluid processing apparatuses (FPAs) used in the STS-115 and STS-123 experiments. Panel A: Schematic diagram of an FPA. An FPA consists of a glass barrel that contains a short bevel on one side and stoppers inside that separate individual chambers containing fluids used in the experiment. The glass barrel loaded with stoppers and fluids is housed inside a lexan sheath containing a plunger that pushes on the top stopper to facilitate mixing of fluids at the bevel. The bottom stopper in the glass barrel (and also the bottom of the lexan sheath) is designed to contain a gas-permeable membrane that allows air exchange during bacterial growth. In the STS-115 and STS-123 experiments, the bottom chamber contained media, the middle chamber contained the bacterial inoculum suspended in PBS, and the top chamber contained either RNA/protein fixative or additional media. Upon activation, the plunger was pushed down so that only the middle chamber fluid was mixed with the bottom chamber to allow media inoculation and bacterial growth. At this step, the plunger was pushed until the bottom of the middle rubber stopper was at the top part of the bevel. After the 25-hour growth period, the plunger was pushed until the bottom of the top rubber stopper was at the top part of the bevel such that the top chamber fluid was added. Panel B: Photograph of FPAs in pre-flight configuration. Panel C: Photograph of FPAs in post-flight configuration showing that all stoppers have been pushed together and the entire fluid sample is in the bottom chamber.
(0.05 MB PDF)

Figure S4 Primers used in this study for qRT-PCR.
(0.03 MB PDF)

Table S1 Salmonella typhimurium proteins identified via MudPit analysis as present during growth in M9 minimal media in spaceflight (173 proteins total). Proteomic analysis of Salmonella typhimurium cultured in M9 media during spaceflight.
(0.12 MB PDF)
