# Supplementary figures and images for: Media Ion Composition Controls Regulatory and Virulence Response of Salmonella in Spaceflight
Source: PLoS One. 2008 Dec 12;3(12):e3923. doi: 10.1371/journal.pone.0003923 (PMC2592540; doi:10.1371/journal.pone.0003923)

**A**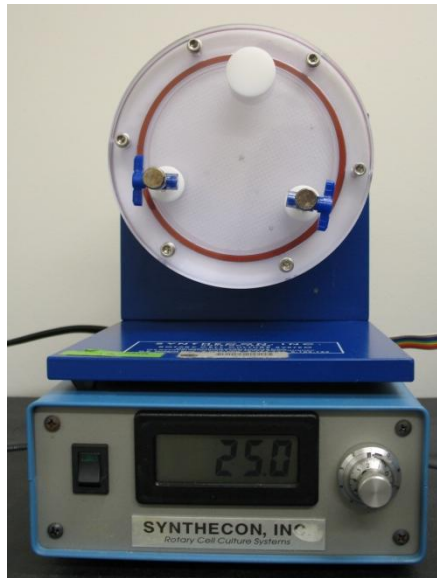**B**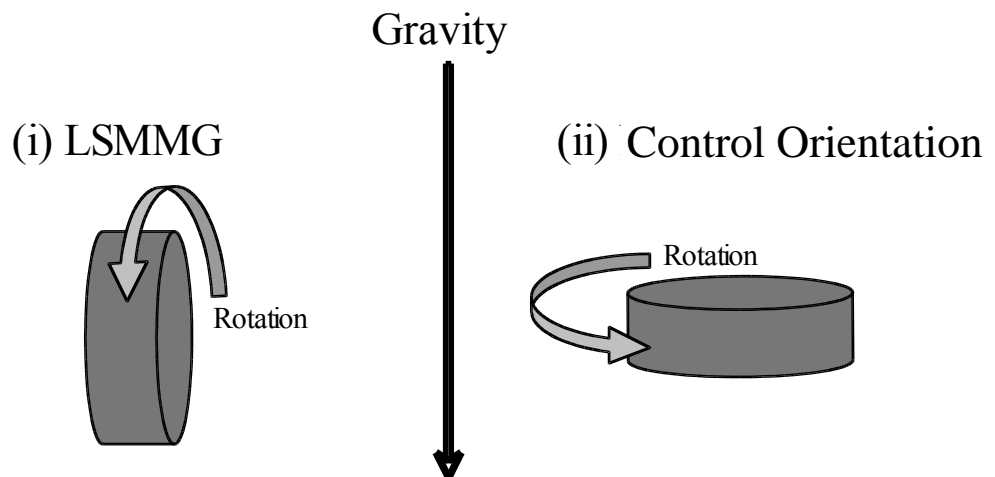**C**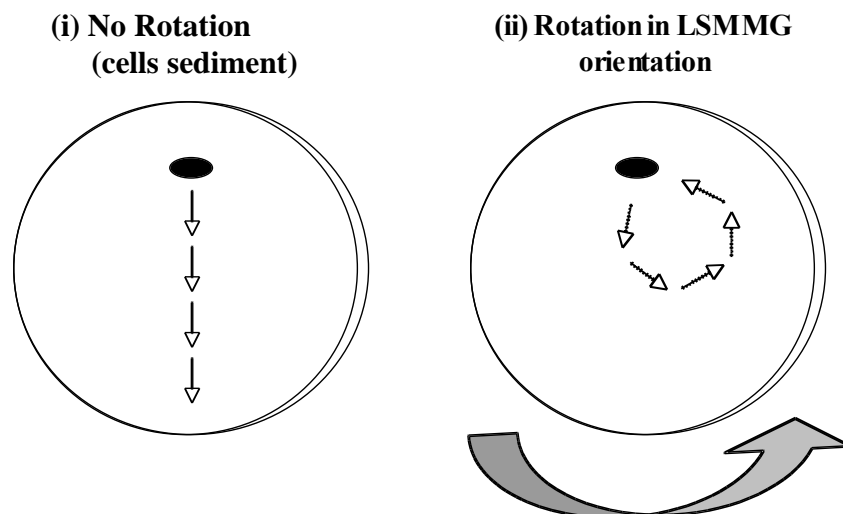

Supplement: Figure S1 — The rotating wall vessel (RWV) bioreactor and power supply. Panel A: The cylindrical culture vessel is completely filled with culture medium through ports on the face of the vessel and operates by rotating around a central axis. Cultures are aerated through a hydrophilic membrane that covers the back of the cylinder. The power supply is shown below the bioreactor. Panel B: The two operating orientations of the RWV are depicted. In the LSMMG orientation (panel i), the axis of rotation of the RWV is perpendicular to the direction of the gravity force vector. In the control orientation (panel ii), the axis of rotation is parallel with the gravity vector. Panel C: The effect of RWV rotation on particle suspension is depicted. When the RWV is not rotating, or rotating in the control orientation (panel i), the force of gravity will cause particles in apparatus to sediment and eventually settle on the bottom of the RWV. When the RWV is rotating in the LSMMG position (panel ii), particles are continually suspended in the media. The media within the RWV rotates as a single body, and the sedimentation of the particle due to gravity is offset by the upward forces of rotation. The result is low shear aqueous suspension that is strikingly similar to what would occur in true microgravity. (0.15 MB PDF) [file pone.0003923.s001.pdf]

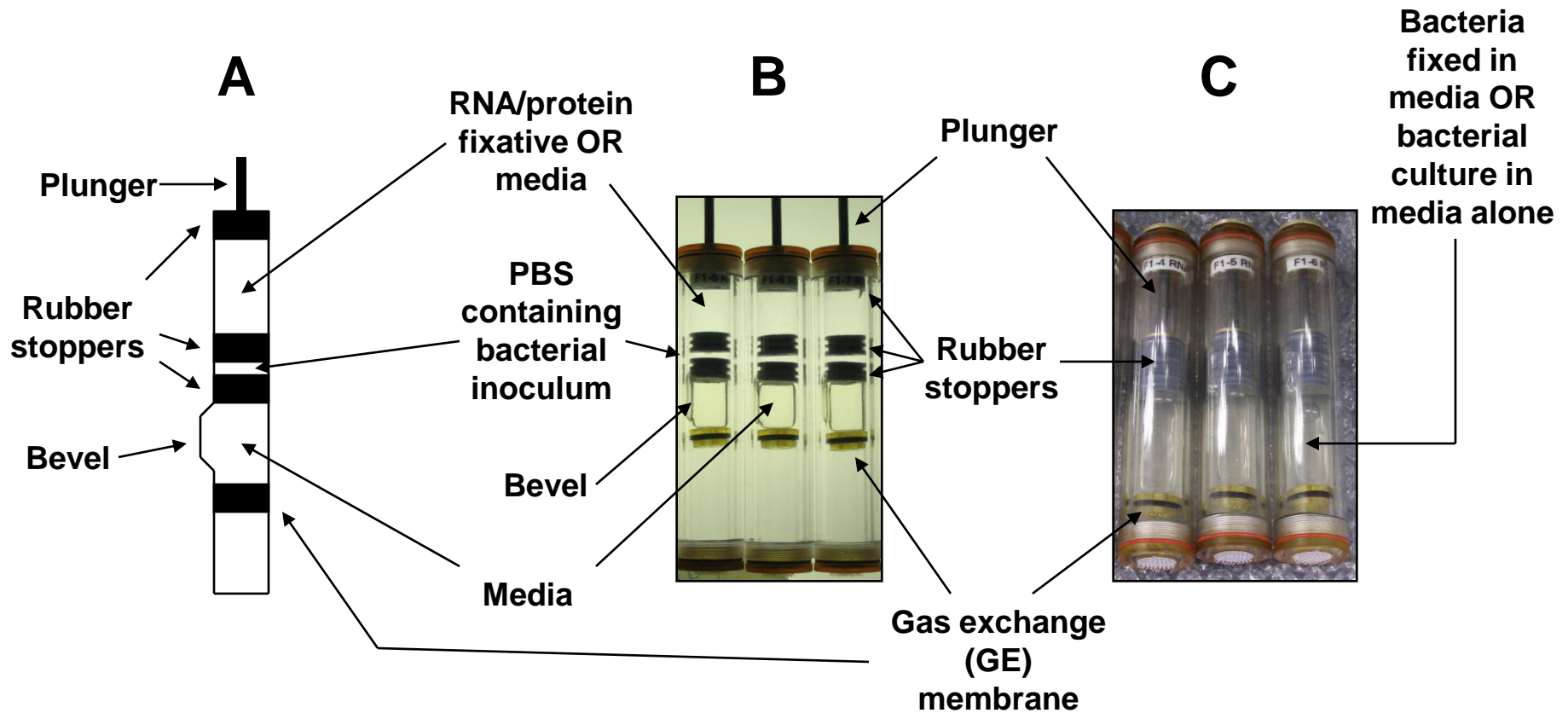

Supplement: Figure S3 — Diagram and photographs of fluid processing apparatuses (FPAs) used in the STS-115 and STS-123 experiments. Panel A: Schematic diagram of an FPA. An FPA consists of a glass barrel that contains a short bevel on one side and stoppers inside that separate individual chambers containing fluids used in the experiment. The glass barrel loaded with stoppers and fluids is housed inside a lexan sheath containing a plunger that pushes on the top stopper to facilitate mixing of fluids at the bevel. The bottom stopper in the glass barrel (and also the bottom of the lexan sheath) is designed to contain a gas-permeable membrane that allows air exchange during bacterial growth. In the STS-115 and STS-123 experiments, the bottom chamber contained media, the middle chamber contained the bacterial inoculum suspended in PBS, and the top chamber contained either RNA/protein fixative or additional media. Upon activation, the plunger was pushed down so that only the middle chamber fluid was mixed with the bottom chamber to allow media inoculation and bacterial growth. At this step, the plunger was pushed until the bottom of the middle rubber stopper was at the top part of the bevel. After the 25-hour growth period, the plunger was pushed until the bottom of the top rubber stopper was at the top part of the bevel such that the top chamber fluid was added. Panel B: Photograph of FPAs in pre-flight configuration. Panel C: Photograph of FPAs in post-flight configuration showing that all stoppers have been pushed together and the entire fluid sample is in the bottom chamber. (0.05 MB PDF) [file pone.0003923.s003.pdf]
