# Supplementary material for: Media Ion Composition Controls Regulatory and Virulence Response of Salmonella in Spaceflight
Source: PLoS One. 2008 Dec 12;3(12):e3923. doi: 10.1371/journal.pone.0003923 (PMC2592540; doi:10.1371/journal.pone.0003923)
Supplement: Figure S2 — Experimental setup for STS-115 and STS-123 Salmonella typhimurium microarray and virulence experiments. This flowchart displays a timeline of how the STS-115 and STS-123 experiments were designed and organized. Fluid processing apparatuses (FPAs) were loaded as in Supplemental Figure 3 and delivered to Shuttle, activated during spaceflight, and recovered upon landing as outlined in the flowchart. For a more detailed description of the FPA activation and fixation/supplementation steps, please refer to Supplemental Figure 3. OES: Orbital Environmental Simulator (this is a climate-controlled room at Kennedy Space Center that houses ground controls and is maintained at the same temperature and humidity as the Space Shuttle via real-time communications). SLSL: Space Life Sciences Lab. (0.01 MB PDF) [file pone.0003923.s002.pdf]

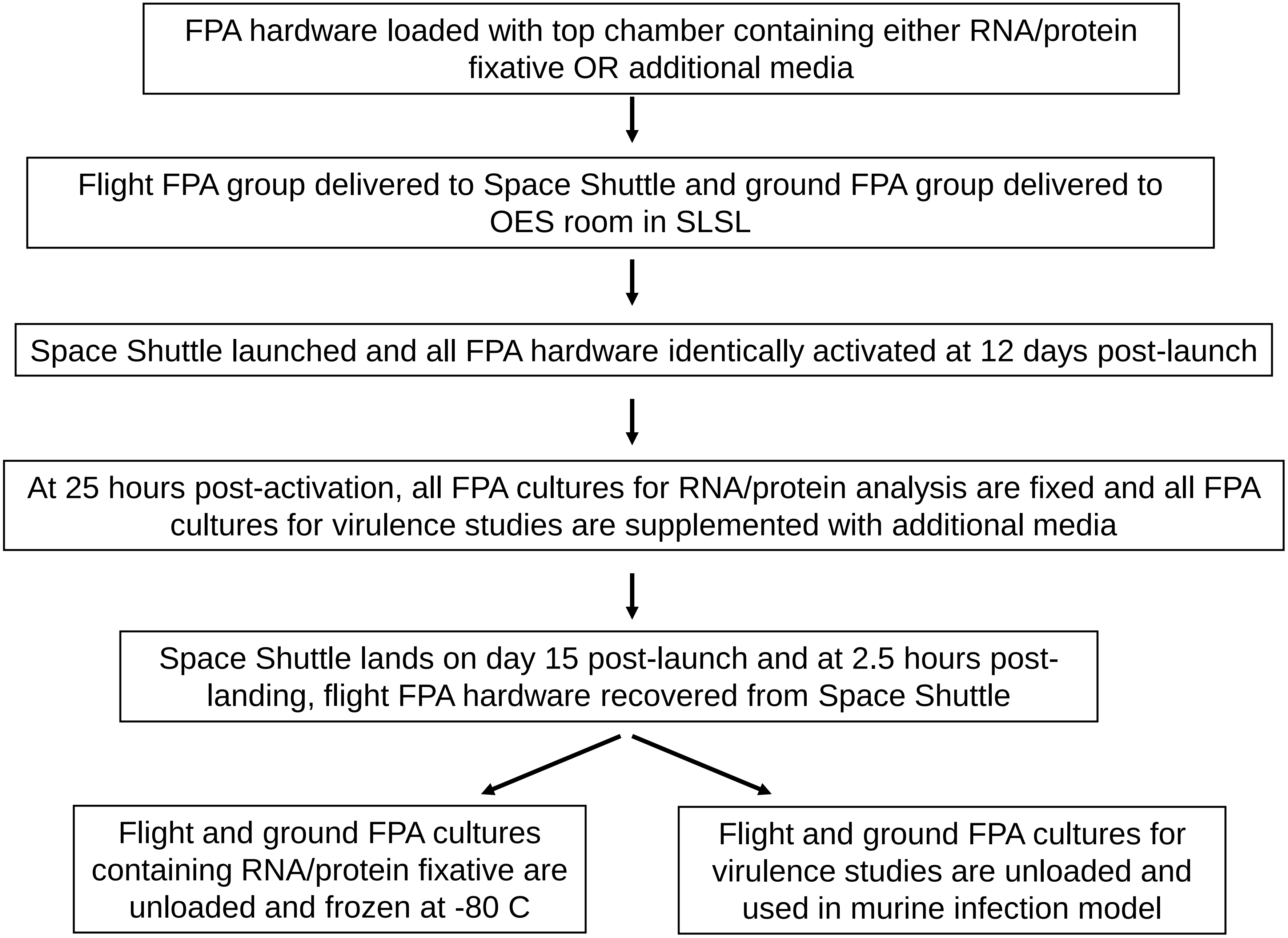
FPA hardware loaded with top chamber containing either RNA/protein fixative OR additional media
Flight FPA group delivered to Space Shuttle and ground FPA group delivered to OES room in SLSL
Space Shuttle launched and all FPA hardware identically activated at 12 days post-launch
At 25 hours post-activation, all FPA cultures for RNA/protein analysis are fixed and all FPA cultures for virulence studies are supplemented with additional media
Space Shuttle lands on day 15 post-launch and at 2.5 hours post-landing, flight FPA hardware recovered from Space Shuttle
Flight and ground FPA cultures containing RNA/protein fixative are unloaded and frozen at -80 C
Flight and ground FPA cultures for virulence studies are unloaded and used in murine infection model
