# Supplementary material for: Media Ion Composition Controls Regulatory and Virulence Response of Salmonella in Spaceflight
Source: PLoS One. 2008 Dec 12;3(12):e3923. doi: 10.1371/journal.pone.0003923 (PMC2592540; doi:10.1371/journal.pone.0003923)
Supplement: Figure S4 — Primers used in this study for qRT-PCR. (0.03 MB PDF) [file pone.0003923.s004.pdf]

Supplemental Figure 4. Primers used in this study for qRT-PCR.

5Sal16S

gtaacggctcaccaaggcgacgatccctag

Sal16S3

cttcgccaccggtattcctccagatctctac

5STM1724 (for *trpD*)

agcgcctttgtcgcggcggcctgtgga

STM17243 (for *trpD*)

gttgatcagcgggccgagtacgttgaacag

5rnpB

gtcgtggacagtcattcatctaggccagca

rnpB3

ctccatagggcagggtgccaggtaacgcct

5csrB

tttcctgtgaccttacggcctgttcatcctg

csrB3

agcaggacacgccaggatggtgttacaagg

5yfiD

tacgagcgataacgtcgcgctgctgttccg

yfiD3

gctgaattccttctggctgctggacagcga
