# Supplementary material for: Media Ion Composition Controls Regulatory and Virulence Response of Salmonella in Spaceflight
Source: PLoS One. 2008 Dec 12;3(12):e3923. doi: 10.1371/journal.pone.0003923 (PMC2592540; doi:10.1371/journal.pone.0003923)
Supplement: Table S1 — Salmonella typhimurium proteins identified via MudPit analysis as present during growth in M9 minimal media in spaceflight (173 proteins total). Proteomic analysis of Salmonella typhimurium cultured in M9 media during spaceflight. (0.12 MB PDF) [file pone.0003923.s005.pdf]

Protein name	Accession number	Protein molecular weight (Daltons)	Flight total cell protein ID probability*	Ground total cell protein ID probability*
sn-glycerol-3-phosphate dehydrogenase	gi|16766813	57 kDa	100% (100%)	99% (99%)
branched-chain-amino-acid transaminase	gi|96710	34 kDa	99% (99%)	100% (100%)
putative periplasmic protein	gi|16764930	39 kDa	100% (100%)	100% (100%)
6,7-dimethyl-8-ribityllumazine synthase	gi|16501685	16 kDa	100% (100%)	100% (100%)
thioredoxin reductase	gi|16502122	35 kDa	100% (100%)	100% (100%)
50S ribosomal subunit protein L2	gi|16505152	30 kDa	100% (100%)	100% (100%)
30S ribosomal protein S10	gi|68057571	12 kDa	100% (100%)	100% (100%)
50S ribosomal protein L24	gi|15803836	11 kDa	100% (100%)	100% (100%)
serine hydroxymethyltransferase	gi|16503768	45 kDa	100% (100%)	100% (100%)
30s ribosomal protein S6	gi|16505516	15 kDa	100% (100%)	100% (100%)
30S ribosomal protein S22	gi|16502603	5 kDa	100% (100%)	100% (100%)
2,3,4,5-tetrahydropyridine-2-carboxylate N-succinyltransferase	gi|16763603	30 kDa	100% (100%)	100% (100%)
peptidyl-prolyl cis-trans isomerase B	gi|16501803	18 kDa	100% (100%)	100% (100%)
FKBP-type peptidyl-prolyl cis-trans isomerase	gi|16766744	21 kDa	100% (100%)	100% (100%)
ribosome recycling factor	gi|16501500	21 kDa	100% (100%)	100% (100%)
carbamoyl-phosphate synthase large subunit	gi|16763457	118 kDa	100% (100%)	100% (100%)
ATP synthase delta subunit	gi|16504763	19 kDa	100% (100%)	100% (100%)
FKBP-type peptidyl-prolyl cis-trans isomerase	gi|16766742	29 kDa	99% (99%)	100% (100%)
putative cytoplasmic protein	gi|56383221	12 kDa	99% (99%)	100% (100%)
D-3-phosphoglycerate dehydrogenase	gi|16766363	44 kDa	100% (100%)	100% (100%)
50S ribosomal subunit protein L3	gi|16505149	22 kDa	100% (100%)	100% (100%)
ATP synthase alpha subunit	gi|16504764	55 kDa	100% (100%)	100% (100%)
serine endoprotease	gi|16766643	47 kDa	100% (100%)	100% (100%)
putative ABC-type transport system ATPase	gi|16763890	30 kDa	99% (99%)	100% (100%)
ATP synthase subunit B	gi|16504762	17 kDa	99% (99%)	100% (100%)
malate dehydrogenase	gi|16766654	32 kDa	99% (99%)	100% (100%)
RNase E	gi|16764541	119 kDa	100% (100%)	100% (100%)
osmotically inducible protein C	gi|16502605	15 kDa	99% (99%)	100% (100%)
polynucleotide phosphorylase	gi|16766580	77 kDa	100% (100%)	100% (100%)
glutamate dehydrogenase	gi|16764650	49 kDa	100% (100%)	100% (100%)
menaquinone biosynthesis protein	gi|16504650	17 kDa	99% (99%)	100% (100%)
50S ribosomal subunit protein L18	gi|16505166	13 kDa	99% (99%)	100% (100%)
phospho-2-dehydro-3-deoxyheptonate aldolase	gi|16503824	39 kDa	99% (99%)	100% (100%)
transketolase	gi|16766377	72 kDa	99% (99%)	100% (100%)
acetylglutamate kinase	gi|16767387	27 kDa	99% (99%)	100% (100%)
50S ribosomal protein L11	gi|15804573	15 kDa	99% (99%)	100% (100%)
cold shock protein CspC	gi|15802236	7 kDa	100% (100%)	100% (100%)
unnamed protein product	gi|47736	16 kDa	100% (100%)	100% (100%)
dihydrolipoamide dehydrogenase	gi|16763544	51 kDa	100% (100%)	100% (100%)
50S ribosomal subunit protein L29	gi|16505157	7 kDa	100% (100%)	100% (100%)
arginine-binding periplasmic protein 1 precursor	gi|16502093	27 kDa	100% (100%)	100% (100%)
50S ribosomal subunit protein L10	gi|47916	18 kDa	100% (100%)	100% (100%)
hyperosmotically-inducible periplasmic protein	gi|16505665	21 kDa	100% (100%)	100% (100%)
bacterioferritin comigratory protein	gi|16503708	18 kDa	100% (100%)	100% (100%)
glutamate/aspartate transporter	gi|16764042	34 kDa	100% (100%)	100% (100%)
50S ribosomal subunit protein L17	gi|16505176	14 kDa	100% (100%)	100% (100%)
50S ribosomal subunit protein L6	gi|16505165	19 kDa	100% (100%)	100% (100%)
fructose 1,6-bisphosphate aldolase	gi|16504152	39 kDa	100% (100%)	100% (100%)
30S ribosomal protein S3	gi|16131193	26 kDa	100% (100%)	100% (100%)
5-methyltetrahydropteroyltriglutamate--homocysteine methyltransferase	gi|16767235	85 kDa	100% (100%)	100% (100%)
phosphate acetyltransferase	gi|16765665	77 kDa	99% (99%)	100% (100%)
inorganic pyrophosphatase	gi|16505542	20 kDa	100% (100%)	100% (100%)
phase 1 flagellin	gi|50830926	52 kDa	99% (99%)	100% (100%)
histone like DNA-binding protein HU-alpha (NS2) (HU-2)	gi|16504591	10 kDa	100% (100%)	100% (100%)
Iron transport protein, periplasmic-binding protein	gi|16503939	34 kDa	100% (100%)	100% (100%)
conserved hypothetical protein	gi|16503804	14 kDa	100% (100%)	100% (100%)
ribosomal protein S7	gi|47922	18 kDa	100% (100%)	100% (100%)
ATP synthase beta subunit	gi|16504766	50 kDa	100% (100%)	100% (100%)
30S ribosomal protein S2	gi|16501497	27 kDa	100% (100%)	100% (100%)
glucose-specific PTS system enzyme IIA component	gi|47658	18 kDa	100% (100%)	100% (100%)
probable peroxidase	gi|16501671	22 kDa	100% (100%)	100% (100%)
50S ribosomal subunit protein L5	gi|16505162	20 kDa	100% (100%)	100% (100%)
argininosuccinate lyase	gi|16767388	50 kDa	100% (100%)	100% (100%)
RNA polymerase, alpha subunit	gi|24053769	37 kDa	100% (100%)	100% (100%)
glycine/betaine/proline transport protein	gi|16766122	36 kDa	100% (100%)	100% (100%)
elongation factor Ts	gi|16501498	30 kDa	100% (100%)	100% (100%)
inositol-5-monophosphate dehydrogenase	gi|16765831	52 kDa	100% (100%)	100% (100%)
putative outer membrane lipoprotein	gi|16763634	29 kDa	100% (100%)	100% (100%)
DNA-directed RNA polymerase, beta-subunit	gi|16504603	155 kDa	100% (100%)	100% (100%)
GroEL protein	gi|16505460	57 kDa	100% (100%)	100% (100%)
enolase	gi|16504025	46 kDa	100% (100%)	100% (100%)
glyceraldehyde 3-phosphate dehydrogenase A	gi|16502901	36 kDa	100% (100%)	100% (100%)
translation elongation factor EF-Tu.A	gi|96718	43 kDa	100% (100%)	100% (100%)
iron-dependent alcohol dehydrogenase	gi|16765093	96 kDa	100% (100%)	100% (100%)
phosphoglycerate kinase	gi|16504153	41 kDa	100% (100%)	100% (100%)
putative hydrogenase membrane component precurosr	gi|16764429	38 kDa	100% (100%)	100% (100%)
formate acetyltransferase 1	gi|16502136	85 kDa	100% (100%)	100% (100%)
30S ribosomal protein S1	gi|16502144	61 kDa	100% (100%)	100% (100%)
elongation factor G	gi|47923	78 kDa	100% (100%)	100% (100%)
30S ribosomal subunit protein S5	gi|24053777	18 kDa	100% (100%)	100% (100%)
O-Acetylserine Sulfhydrylase	gi|11514514	34 kDa	100% (100%)	100% (100%)
trigger factor	gi|16501718	48 kDa	100% (100%)	100% (100%)
Glutamine Synthetase	gi|9256972	52 kDa	100% (100%)	100% (100%)
molecular chaperone DnaK	gi|16763402	69 kDa	100% (100%)	100% (100%)
arginine-binding periplasmic protein 2 precursor	gi|16502090	27 kDa	100% (100%)	100% (100%)
alkyl hydroperoxide reductase c22 protein	gi|16501859	21 kDa	100% (100%)	100% (100%)

50S ribosomal protein L9	gi|16767640	16 kDa	100% (100%)	100% (100%)
outer membrane protein OmpH precursor	gi|16501506	18 kDa	100% (100%)	100% (100%)
glutamine-binding periplasmic protein precursor	gi|16502041	27 kDa	100% (100%)	100% (100%)
GroES protein	gi|16505459	10 kDa	100% (100%)	100% (100%)
50S ribosomal subunit protein L1	gi|16504607	25 kDa	100% (100%)	100% (100%)
outer membrane protein C	gi|16503494	41 kDa	100% (100%)	100% (100%)
dipeptide transport protein	gi|16766917	60 kDa	100% (100%)	
PTS system protein HPr	gi|24052838	9 kDa	100% (100%)	
50S ribosomal subunit protein L7/L12	gi|47917	12 kDa	100% (100%)	
50S ribosomal subunit protein L32	gi|24051382	6 kDa	100% (100%)	
oligopeptide transport protein	gi|39546324	61 kDa	100% (100%)	
high-affinity branched-chain amino acid transporter	gi|16766853	39 kDa	100% (100%)	
30S ribosomal protein S20	gi|16501327	10 kDa	100% (100%)	
phosphoribosylaminoimidazole carboxylase catalytic subunit	gi|16763914	18 kDa	100% (100%)	
putative translation initiation inhibitor	gi|16505567	14 kDa	100% (100%)	
putative multicopper oxidase	gi|16763558	59 kDa	100% (100%)	
DNA protection during starvation protein	gi|16502042	19 kDa	100% (100%)	
thioredoxin	gi|67005950	12 kDa	100% (100%)	
sulfate transport protein	gi|16767329	37 kDa	100% (100%)	
ribulose-phosphate 3-epimerase	gi|16766771	24 kDa	99% (99%)	
cytoplasmic ferritin	gi|16765276	19 kDa	99% (99%)	
osmotically inducible lipoprotein E precursor	gi|16502880	12 kDa	100% (100%)	
fructose-bisphosphate aldolase class I	gi|16503381	38 kDa	100% (100%)	
30S ribosomal protein S13	gi|16766707**	13 kDa	100% (100%)	
thiosulfate transport protein	gi|16765764	38 kDa	100% (100%)	
histidine-binding periplasmic protein	gi|47731	28 kDa	100% (100%)	
RecA protein	gi|16503906	38 kDa	100% (100%)	
aspartate semialdehyde dehydrogenase	gi|2353187	43 kDa	100% (100%)	
transcription elongation factor NusA	gi|16766585**	55 kDa	100% (100%)	
pyruvate kinase	gi|16764728	51 kDa	99% (99%)	
30S ribosomal protein S11	gi|16766706**	14 kDa	99% (99%)	
DNA-directed RNA polymerase omega subunit	gi|15804190	10 kDa	99% (99%)	
single-strand DNA-binding protein	gi|16505243	19 kDa	99% (99%)	
DNA-binding protein HU-beta	gi|581767	9 kDa	99% (99%)	
putative cytoplasmic protein	gi|6851082	19 kDa	99% (99%)	
succinyl-CoA synthetase beta chain	gi|16501970	41 kDa	99% (99%)	
50S ribosomal subunit protein A	gi|56383471	7 kDa	99% (99%)	
ATPase subunit	gi|7594817	46 kDa	99% (99%)	
ketol-acid reductoisomerase	gi|16767185	54 kDa	98% (98%)	
DNA ligase	gi|16765747	73 kDa	99% (99%)	
30S ribosomal subunit protein S19	gi|16505153	10 kDa	100% (100%)	
NifU-like protein involved in Fe-S cluster formation	gi|16503756	14 kDa	99% (99%)	
putative sigma(54) modulation protein	gi|16503819	13 kDa	99% (99%)	
3-isopropylmalate dehydrogenase	gi|16763502**	40 kDa	99% (99%)	
bacterioferrin	gi|16766732**	18 kDa	99% (99%)	
glycerate kinase II	gi|16763905**	39 kDa	99% (99%)	
DNA polymerase I	gi|16767264	103 kDa	99% (99%)	
putative periplasmic protein	gi|16764812	54 kDa	99% (99%)	
threonine dehydratase	gi|16767181	56 kDa	98% (98%)	
putative universal stress protein UspA	gi|16501866	16 kDa	99% (99%)	
ferric uptake regulator Fur	gi|16501929	17 kDa	99% (99%)	
Cell division protease ftsH	gi|16504361	71 kDa	99% (99%)	
flavodoxin	gi|16764064	20 kDa	99% (99%)	
nitrate reductase 2 beta subunit	gi|16764922	59 kDa	99% (99%)	
keto-hydroxyglutarate-aldolase/keto-deoxy-phosphogluconate aldolase	gi|16765226	22 kDa	99% (99%)	
carbon storage regulator CsrA	gi|24053109	7 kDa	99% (99%)	
rmlC dTDP-4,deoxyrhamnose 3,5 epimerase	gi|581655	21 kDa	99% (99%)	
TonB-dependent siderophore receptor protein	gi|16766089	79 kDa		100% (100%)
N-succinyldiaminopimelate-aminotransferase/acetylornithine transaminase	gi|16766756	44 kDa		100% (100%)
DNA-directed RNA polymerase beta subunit	gi|16767407	151 kDa		100% (100%)
argininosuccinate synthase	gi|39546365	50 kDa		100% (100%)
outer membrane lipoprotein SlyB precursor	gi|16502764	16 kDa		100% (100%)
thiol peroxidase	gi|16765025	18 kDa		100% (100%)
50S ribosomal subunit protein L14	gi|49613467	14 kDa		100% (100%)
triosephosphate isomerase	gi|16767347	27 kDa		100% (100%)
lipoprotein	gi|16765808**	37 kDa		100% (100%)
oligopeptidase A	gi|16766880	77 kDa		100% (100%)
phosphoribosylamine--glycine ligase	gi|16767429	46 kDa		100% (100%)
phosphoglyceromutase	gi|16764136	28 kDa		100% (100%)
cystathionine gamma-synthase	gi|16767366	42 kDa		100% (100%)
glucose-6-phosphate isomerase	gi|16767471	61 kDa		100% (100%)
50S ribosomal subunit protein L33	gi|24054147	6 kDa		100% (100%)
ribosomal protein S4	gi|2780215	23 kDa		100% (100%)
PEP-protein phosphotransferase	gi|16765752**	63 kDa		100% (100%)
Lpp1 major outer membrane lipoprotein	gi|37785814	8 kDa		100% (100%)
aldose 1-epimerase	gi|16764640**	33 kDa		100% (100%)
putative outer membrane porin precursor	gi|16764916**	40 kDa		99% (99%)
50S ribosomal protein L4	gi|15803846	22 kDa		100% (100%)
outer membrane ferric enterobactin receptor precursor	gi|16763962	83 kDa		100% (100%)
hypothetical protein STM2795 putative LysM domain	gi|16766106**	16 kDa		100% (100%)
phosphoribosylaminoimidazole-succinocarboxamide synthase	gi|16503704	27 kDa		100% (100%)
arnithine carbamoyltransferase	gi|312706	24 kDa		100% (100%)
ribonucleoside-diphosphate reductase	gi|1184247	81 kDa		100% (100%)
biotin carboxylase	gi|16504442	49 kDa		100% (100%)
3-ketoacyl-(acyl-carrier-protein) reductase	gi|16764550	26 kDa		100% (100%)
6-phosphofructokinase II	gi|16764677	33 kDa		100% (100%)
uroporphyrinogen III methylase	gi|16767207	42 kDa		100% (100%)

*Peptide samples obtained from MudPIT were analyzed using Sequest and X!Tandem software, and the data was organized using the Scaffold program. To be considered a positive identification in Scaffold, the following parameters were used: a minimum of 2 peptides from a given protein identified with peptide and protein thresholds of 80% to give an overall protein identification (ID) probability of at least 80%. Note that a protein ID probability of greater than 80% in at least one of the samples warranted inclusion in the table so as to allow identification of possible differential expression of a given protein.

** Peptides encoded by genes or operons also found to be differentially regulated in spaceflight or ground based modeled microgravity in LB medium
